# Engineering, Recyclable, and Biodegradable Plastics in the Automotive Industry: A Review

**DOI:** 10.3390/polym14163412

**Published:** 2022-08-21

**Authors:** Horacio Vieyra, Joan Manuel Molina-Romero, Juan de Dios Calderón-Nájera, Alfredo Santana-Díaz

**Affiliations:** 1 Tecnologico de Monterrey, Escuela de Ingeniería y Ciencias, Eduardo Monroy Cárdenas 2000, San Antonio Buenavista, Toluca de Lerdo 50110, Mexico; 2 Tecnologico de Monterrey, Escuela de Ingeniería y Ciencias, Eugenio Garza Sada 2501, Monterrey 64849, Mexico

**Keywords:** automotive industry, engineering plastic, recyclable, biodegradable, bioplastics, lightweight, cars, polymers

## Abstract

The automotive industry has used plastics almost since the beginning. The lightness, flexibility, and many qualities of plastics make them ideal for the automotive industry, reducing cars’ overall weight and fuel consumption. Engineering plastics in this industry belong to the high-performance segment of non-renewable resources. These plastics exhibit higher properties than commodity plastics. Fortunately, unlike recycled commodity plastics, the super properties and high-performance characteristics make engineering plastics effectively reused after recycling. The substitution of these fossil-fuel-derived plastics adds to the solution of lightweighting, a much-needed solution to waste management, and solves industrial and ecological issues surrounding plastic disposal. All major vehicle manufacturers worldwide use bioplastics and bio-based plastics, including natural-fiber composites and engineering plastics reinforced with natural fibers. Changing the source of plastics to raw materials from renewable resources is the logical approach to sustainability. Thus, high-quality plastics, recycled plastics, bio-based plastics, and biodegradable plastics could be exploited from design, making sustainability an integral concept of mobility development. This review analyzes that switching from fossil-fuel- to renewable-sources-derived plastics is a step toward meeting the current environmental goals for the automotive industry, including electric cars.

## 1. Introduction

The automotive industry is critical to economics, research and development, and global innovation growth. The global automotive industry is an essential source of employment, accounting for over 5% of the world’s total manufacturing employees, with nearly 14 million workers. It is the source of 11.5% of the global turnover (gross revenue), making over 66 million cars (including vans, trucks, and buses) per year which represents an output of almost EUR 2 trillion [1,2].

According to the International Energy Agency (IEA) 2019 report, passenger cars (including cars, sport utility vehicles, and personal trucks) alone use more energy for their operation than the whole residential sector and use comparatively the same energy as the entire manufacturing industry [3]. The transportation sector accounts for 20.5% of global energy consumption [4].

The operational energy consumption in vehicles is directly related to weight, and weight is directly related to the materials. It is important to have lighter materials to reduce energy consumption, but it is also essential that these materials are friendly to the environment. The transport sector accounted for almost a third of the final energy-related CO_2_ emissions, of which 72% comes from road transportation, mostly from passenger cars [5]. The large amounts of energy consumed are also the source of the large quantities of CO_2_ produced by the automotive industry [6,7].

Vehicle fuel consumption results from moving the mass of the vehicle and other losses (e.g., aerodynamic drag, accessories, engine, and powertrain friction) [8,9]. Because up to 50% of a wheeled vehicle’s fuel consumption is mass-dependent, vehicle lightweighting provides the opportunity to reduce use-phase fuel consumption [10]. Fuel economy regulations have helped drive technological advancements to mitigate use-phase impacts of individual vehicles by improving powertrains (electric and hybrid high-efficiency internal combustion) [11,12] and reducing vehicle mass (lightweighting) [13], [14]. There is a strong tendency to achieve lightweighting through material substitution and changes in design and construction. Thus, plastics typically make up 18% of a new vehicle’s average weight, with more than 50% of a modern vehicle’s volume [15].

Together with environmental bodies, governments are encouraging sustainable and eco-efficient materials using policies targeting waste generation, recycling, and carbon emission. Four areas require high-priority lightweighting research and development with plastics: interior, body, powertrain, and chassis. Additionally, bioplastics and composites are good candidates to substitute metals and metal alloys to manufacture several automotive components. This review highlights the importance of plastics in reducing weight, energy consumption, or CO_2_ emissions, but above all, the need for sustainable plastics in the automotive industry. In this review, we first describe the current use of plastics in the automotive industry, particularly those produced from non-renewable sources, and we comment on the potential use of biodegradable, recyclable, and reusable polymers. Furthermore, we discuss the automotive parts currently made from recyclable and renewable-resources plastics, and we also address the new biomaterials with the most significant potential for this industry. When addressing the main environmental issues, we describe the current challenges in waste management and electric vehicles.

## 2. Automotive Plastics

### 2.1. Plastics from Non-Renewable Resources

In the transportation sector, the automotive industry has used plastics almost since the beginning. The lightness, flexibility, and many qualities of plastics make them ideal for the automotive industry, reducing cars’ overall weight and leading to less fuel consumption [16]. Typically, the plastics are used in exterior parts such as the body panels, seal, wheel covers, weatherstripping, bumpers and fender, air dams, trims, and interior features such as the instrument panel, dashboard, door panels, steering wheels, seat and associated parts, instrument panel skin, and decorative pieces. Under hood components such as sensors, ignition compartments, fluid systems, power distribution, and resonators also use plastics [17]. Engineering plastics, such as polyamide (PA), polyphenylene sulfide (PPS), polymethylmethacrylate (PMMA), and acrylonitrile butadiene styrene (ABS), among others, belong to the high-performance segment of non-renewable resources. These plastics exhibit higher properties than commodity plastics, such as polyethylene (PE), polypropylene (PP), polyvinylchloride (PVC), and polystyrene (PS). They exhibit excellent strength, good temperature resistance, toughness, stiffness, chemical resistance, light weight, wear and abrasion resistance, and they easily make automobile components [18]. In some cases, they can easily substitute metal components. The use of engineering plastics goes from single plastics to more sophisticated copolymers, such as the sunroof systems of Renault (a 15% glass-reinforced copolymer compound of styrene maleic anhydride and acrylonitrile butadiene styrene (SMA-GF)) and Citroen (Xiran glass-reinforced blend of styrene maleic anhydride (SMA) and ABS) [19]. The plastics most commonly used in the automotive industry include acrylonitrile butadiene styrene (ABS), polyamide (PA), polycarbonate (PC), polyethylene terephthalate (PET), polystyrene (PS), and polybutylene terephthalate (PBT). Table 1 summarizes the different non-renewable-resource-derived plastics currently used; some have been used in low-volume production [20,21,22,23].

Engineering and conventional plastics, also termed fossil-derived plastics, are not biodegradable by microorganisms within a reasonable time frame. Generally, it would take about 300 years for 60 mm of some plastic films to degrade entirely in soil; this is why plastics are considered an ecological problem [24]. Although capable microorganisms and engineered enzymes make the microbial degradation of polymers such as PET possible [25,26], fossil-derived plastics are not compostable.

### 2.2. Plastics from Renewable Resources

Switching from fossil-fuel- to renewable-sources-derived plastics is a step toward meeting the current environmental goals set for the automotive industry. The substitution of some fossil-fuel-derived plastics adds to the solution of lightweighting, a much-needed solution to waste management, and solves industrial and ecological issues surrounding plastic disposal. Bioplastics, natural-fiber composites, and fiber-reinforced polymer composites are the current alternative. Because there is no consensus on the definitions, we provide descriptions and the extent of their use in automotive applications.

#### 2.2.1. Bioplastics

Bioplastics are a family of bio-based, biodegradable materials, or both. Bio-based plastics are human-made or -processed organic macromolecules from renewable sources such as corn, potatoes, wheat, and vegetable oil through chemical or biological processes [27]. Bio-based plastics, often named biopolymers, are produced by direct extraction from natural substances, the polymerization of monomers derived from biomass, or by microorganisms [28]. Natural biopolymers produced intra- or extra-cellularly in living organisms include cellulose, chitin, starch, polyhydroxyalkanoates (PHA) such as polyhydroxybutyrate (PHB), and synthetic biopolymers built via synthesis reactions, such as polylactide acid (PLA) and others [29].

Bioplastics are biodegradable or compostable. A biodegradable polymer undergoes biodegradation, a chemical process during which microorganisms in the environment decompose materials into natural substances such as water, carbon dioxide, and methane [27]. According to the ASTM international standards 6400 and 6868, compostable plastics must demonstrate proper disintegration during the composting, an adequate level of inherent biodegradation, and no adverse impacts to support plant growth. These standards require that the material biodegrades in a certain period and leaves no toxic residue in the soil. Exposure to the environment (i.e., temperature, moisture, microbial population, pH, and oxygen content) affects the biodegradation of a polymer; thus, a material that degrades by microbial activity under industrial-composting conditions may not degrade in other conditions [30]. Bio-based materials comprise cellulosic plant fibers, bio-based polymers made from monomers from renewable resources, and highly biodegradable polymers.

#### 2.2.2. Cellulosic Plant Fibers

Cellulosic plant fibers are directly isolated from plants such as jute, kenaf, hemp, flax, sisal, banana, bamboo, or coir (coconut fiber) [31]. Renewable plant fibers may replace non-degradable fibers in fiber-reinforced plastic composites for lightweighting [32]. Typical fiber-reinforced plastic composites (such as glass- and carbon-fiber-reinforced plastics) combine the high mechanical and physical performance of the fibers and the appearance and physical properties of the polymers (such as polyester or PP) [31,33]. Fiber-reinforced composites have good mechanical properties per unit weight, are durable, their technologies allow the manufacture of complex and large shapes, and they are useful for crash performance [34]. Unfortunately, the lack of biodegradability of these fiber-reinforced plastics constitutes a significant disadvantage. Thus, substituting industrial fibers with natural fibers is desirable because they have low cost, low environmental impact, and relatively comparable properties to some metals and other composites [35]. Commodity plastics, such as polypropylene and polyethylene, and engineering plastics, such as polycarbonate, polyamides, and polystyrene, are the primary polymer matrix in these composites [36,37]. Another advantage of using natural fibers for composite production is reducing CO_2_ emission [38,39]. When decomposed, the amounts of CO_2_ that the natural fiber releases are the same as what the plant assimilates during the growth phase [40,41].

#### 2.2.3. Highly Biodegradable Polymers

Highly biodegradable polymers (also known as bioengineering polymers, biopolymers, or bioplastics), such as certain aliphatic polyesters (i.e., PHA), starch-based, and protein-based, are highly biodegradable and carbon neutral [42]. Unfortunately, acceptance and widespread applications are not viable due to low production capacity, still-low mechanical properties, and high costs [43]. Highly biodegradable polymers help design eco-friendly products to satisfy customer demand and increasingly strict legislation [44]. However, they are still incapable of many long-term-service-life applications. Most biodegradable products’ applications focus on short-service-life products (i.e., bags, disposable plates and cutlery, packaging materials, and food containers). For automotive applications, newer production technologies to enable the economy of scale are necessary for mass production and must address the challenges of preventing biopolymer degradation during the car’s lifetime.

The National Highway Traffic Safety Administration investigated opportunities for lightweight vehicles using advanced plastics and composites in 2012 [23]. They found that using structural plastics and composites will make lighter, more fuel efficient, and environmentally sustainable vehicles. After modeling and simulation, they concluded that the use of 30% in the content of plastics and composites would have a weight reduction effect without adverse effects on the vehicle crashworthiness. However, bioplastics are still limited in automotive applications primarily because of their low mechanical properties. Additionally, some polymers undergo thermal degradation at low temperatures [45], which means that on a sunny day (where the temperature inside the car reaches 50–70 °C), the polymers could emit an undesirable odor because of their early degradation. Those technical and processable drawbacks hamper their wide-range applicability [46]. Nevertheless, reinforcing helps overcome these drawbacks by blending or adding other polymers or fillers.

Thus, biopolymers can significantly improve the final mechanical properties, thermal behavior, and degradation mechanisms through a cost-effective, accessible, and readily available processing technology [47,48]. The most used bioplastics in the automotive industry include naturally occurring fibers such as soy and hemp, bio-polyamides (bio-PA) and their composites, DuPont Zytel, a combination of nylon resin materials, polylactic acid (PLA), and bio-based polypropylene (bio-PP) [49]. The properties and characteristics of the composite biodegradable material satisfy the increasingly demanding applications that the biodegradable polymer alone could not.

All major vehicle manufacturers worldwide use bioplastics and bio-based plastics, including natural-fiber composites and engineering plastics reinforced with natural fibers such as flax, hemp, jute, and sisal (Table 2). Additionally, Ford vehicles now boast eight sustainable-based materials, including soy foam, wheat straw, kenaf fiber, cellulose, wood, coconut fiber, rice hull, and agave fibers from tequila’s industry waste [50]. Ford is aiming to get rid of single-use plastics by 2030 [51]. Moreover, Toyota’s sustainable development goals towards 2050 include the challenge of establishing a recycling-based society and systems whose primary purpose is to reduce the consumption of dwindling natural resources through the use of renewable resources and recycled materials. The goals are to reduce petroleum-based plastics by developing recycled and eco-plastic technology, meeting quality and performance requirements and establishing collection systems for used plastics [52]. So far, they have researched and expanded the utilization of recycled plastic and collecting and recycling end-of-life bumpers. Audi aims to recycle mixed automotive plastic waste in a resource-conserving closed loop.

### 2.3. Recyclable Plastics

**Figure 1:**
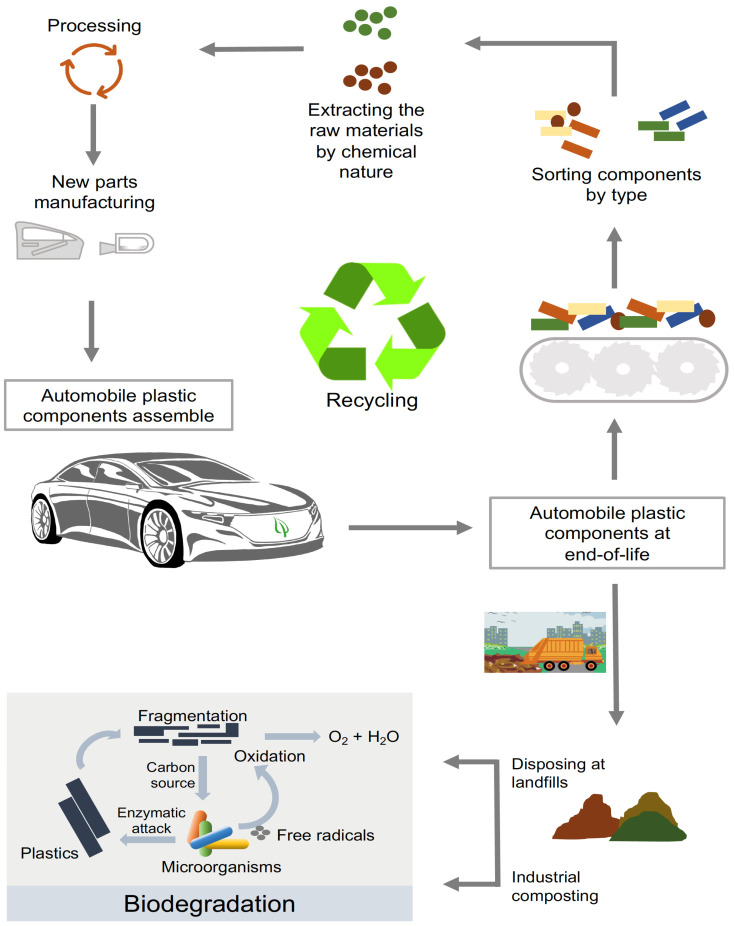
Automobile bioplastic components disposal at end-of-life. Some plastic parts can be recycled, enabling the manufacturer to reuse materials cost-effectively. A plastic disposal program should include one branch of recycling and one of disposing of biodegradable plastics.

The cost increase is one of the critical barriers to using engineering plastics and composites in automobiles. Moreover, extensive usage and rapid growth rates in engineering-plastics’ utilization in automotive applications have resulted in large amounts of plastic waste. Fortunately, unlike recycled-commodity plastics, the super properties and high-performance characteristics make engineering plastics effectively reused after recycling [53]. Using recycled engineering plastics counterbalances the higher price of virgin engineering plastics, which possess superior properties to virgin commodity plastics at a similar cost [54]. Recycling is the most effective management of polymers after their end of life [55]. Recycled engineering plastics must consider the cost of recovering the materials, effective separation of plastic waste, and availability of a proper facility for disassembly, sorting and storing [56]. A good program for the disposal of end-of-life automobiles should include recycling and adequate disposal of engineering plastics and biodegradable plastics. One branch may be dedicated to sorting, extracting, and processing recyclable plastics, and one to composting and disposing of the bioplastics (Figure 1). The recycling branch may produce new automotive parts from the processed materials. The biodegradation branch may reincorporate the material into the soil.

The most common engineering plastics found in automobiles are ABS, PC, polyamides (PA), and polybutylene terephthalate (PBT) [57]. Generally, these materials show good properties after a useful life and can be recycled. Additionally, the toughness of recycled PC significantly increased due to melting blending with maleic anhydride-grafted ABS (ABS-g-MA) [58]. Metal–polymer–metal hybrid sandwiches such as aluminum (Al)-low-density polyethylene (LDPE)-aluminum panels are gaining importance in automotive applications due to their light weight and damping properties. Hybrid materials consisting of metal and thermoplastic parts can be recycled much more efficiently [59]. Moreover, reinforcing recycled plastics may produce new automotive parts with good mechanical properties [60,61].

Recycling bio-based materials produces similar or new products from the materials recovered from sorting, which is advantageous for increasing the material’s lifespan, even more than composting. For instance, using life cycle assessment studies, PLA recycling has a lower environmental impact than composting [62,63]. Compostable plastics are currently not recycled in conventional mechanical-recycling plants due to their low quantity, and mechanical recycling adaptations are still pending [28].

Some recycled plastics are in use in the automotive industry. For example, velour-molded automotive carpets are recycled polyethylene terephthalate (PET) fibers [64]. However, more extraordinary projects are currently underway. The Karlsruhe Institute of Technology (KIT) and Audi launched a pilot project for recycling plastics in 2020. The no-longer-needed plastic parts, such as fuel tanks, decorative wheel trims, or radiator grilles from Audi models, are turned into pyrolysis oil through chemical recycling. The quality of this oil equals that of petroleum products, with the materials made from it offering the same high quality as new goods. The objective is to create new automobile parts from this pyrolysis oil [65]. Ford announced progress towards using 20% renewable and recycled plastics by 2025 [51]. Furthermore, in their 2030 milestone program, Toyota envisions establishing 30 plants for the appropriate treatment and recycling end-of-life vehicles [52].

Overall, the estimated costs, efficiencies, and environmental impact are critical factors when recycling plastics in the automotive industry. Some of the typical plastics used in the industry are relatively cheap to recycle, but they may have a high impact on the environment regarding the carbon footprint. The plastics that release high amounts of CO2 into the environment are unsuitable for recycling. Additionally, the actual fractions of recycled plastic incorporated into the supply chain (efficiency) depend highly on the material class. For example, ABS is easy to recycle, suitable for general use, and highly efficiently recycled (Table 3) [66].

### 2.4. Selecting Plastics for Automotive Applications

**Figure 2:**
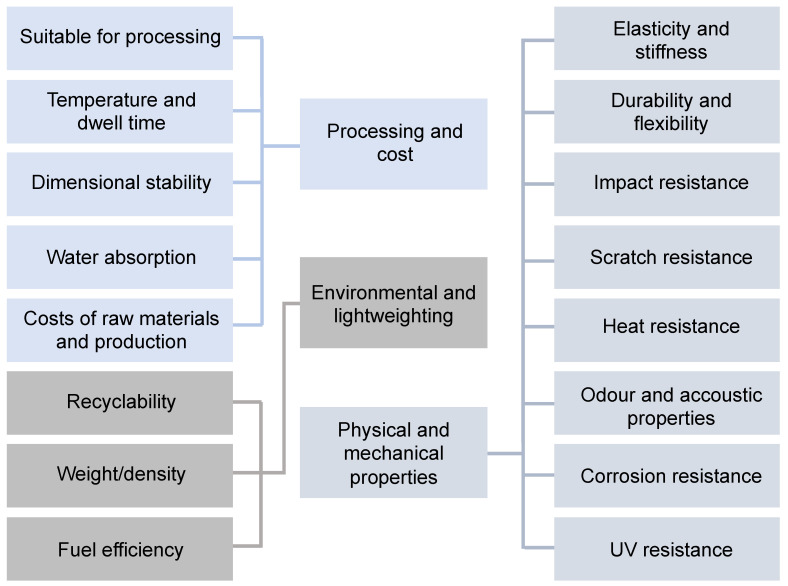
Plastic’s selection criteria for automotive applications.

The selection of materials for automotive applications depends on several criteria that vary according to the type of vehicle or the component type [67,68]. Three main areas require attention while selecting plastics: processing and cost (economic impacts), environment and lightweighting, and physical and mechanical properties (Figure 2). The process and cost aspects relate to the feasibility of manufacturing the components from the selected plastic. Fortunately, manufacturing most bioplastics involves the same technologies currently used for engineering plastics, extrusion, injection molding, injection stretch blow molding, thermoforming, etc. The environment and lightweighting aspects relate to compliance with regulation and legislation on environmental and safety issues. Furthermore, for any robust and tangible application, the mechanical strength of the polymer is the most critical aspect to consider [69].

Mechanical properties indicate the response of a material subjected to different mechanical-loading conditions. Parameters such as tensile strength, impact strength, Young’s modulus, ductility, hardness, plasticity, and yield strengths determine the mechanical strength. The plastic’s performance also depends on time and temperature because of its viscoelastic behavior. Notably, the mechanical properties change when the plastics are painted [70] or blended. However, some biocomposites have successfully retained their mechanical properties. For instance, the lignin has been successfully compatibilized to meet the tensile strength, tensile modulus, and impact characteristics of unfilled composites effectively with load levels of 15% and 25% in high-density polyethylene (HDPE) and PP, respectively [71].

Thermal properties are also very significant to polymeric materials’ potential use in automotive applications. The study of thermal degradation of polymers is essential in designing materials with improved thermal stability and service temperature. Various parameters explain the thermal properties: thermal expansion, heat deflection temperature, critical temperature, glass transition temperature, melting temperature, the heat of fusion, heat of vaporization, flammability, thermal conductivity, and softness [72].

The optical properties of polymers are necessary for a wide range of applications, from packaging to glazing. The optical properties define the interaction of radiation with the material in the visible region. The molecular structure and crystallinity play a significant role in determining optical properties. Analyzing the interaction of radiations with the substances goes from simple visual inspection to more complex methods for determining the optical behavior of the components, such as UV and photoluminescence (PL). Optical parameters such as refractive index, luminous transmittance and haze, photoelastic properties, color, clarity, and gloss determine if a polymer is suitable or not for automotive applications [73].

## 3. Environmental Issues

Manufacturers aim to produce high-performance cars with improved reliability and safety, greater comfort, fuel efficiency, and more competition for environment-concerned users. The requirements for materials that cover the modern automotive industry’s needs are increasingly demanding. However, polyolefins-derived materials remain growing in demand [74]. Plastics will account for more than a third of the growth in petroleum demand by 2030 (3.2 million barrels per day (mb/d)), ahead of road vehicles (2.5 mb/d), aviation (1.7 mb/d), and shipping (0.6 mb/d) [75]. Thus, substituting petrochemical-derived plastics with those made from raw materials from renewable resources may solve both the energy and emissions problems. Unfortunately, substituting petrochemical-derived plastics with biodegradable or recycled ones faces two main challenges that require attention: their contribution to the rapid accumulation of automotive solid waste and plastic litter and the bioplastics production costs. Several strategies are in development to reduce bioplastic costs; for example, molding into components of complex geometries lowers production costs for larger quantities because of its no assembly cost, water-resistant seal, sound absorption, and comfort level (Szeteiova). Additional pending challenges include the ability to dismantle easily.

### 3.1. Candidate Raw Materials from Renewable Resources for the Automotive Industry

**Figure 3:**
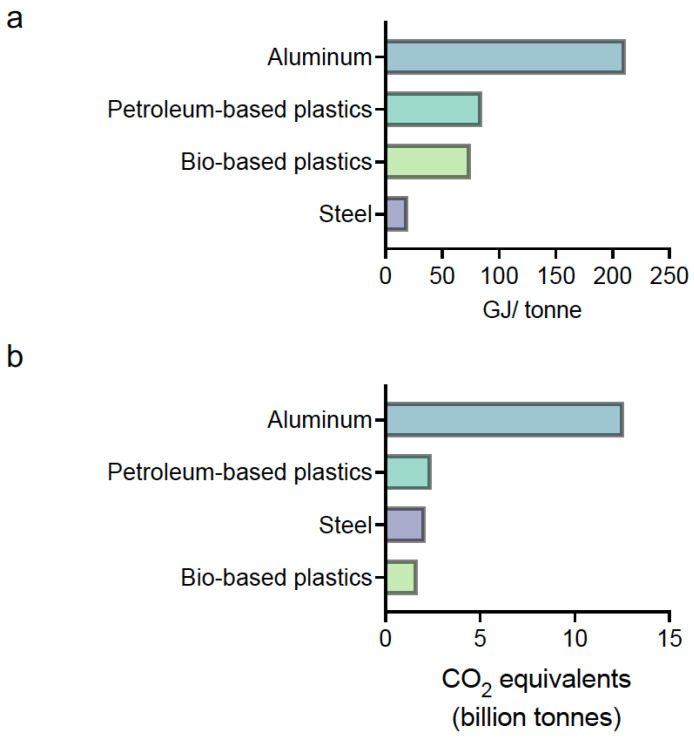
Integrated energy required (**a**) and global CO_2_ emission (**b**) associated with lightweighting materials’ manufacturing.

To reduce ecotoxicity, using raw materials from renewable resources can provide a wide variety of monomers and polymers as ample as those produced by the petrochemical industry [76]. Unlike most commonly used materials, producing bioplastics and bio-based plastics requires less energy. Figure 3a illustrates energy requirements to make 1 ton of the three materials widely used for lightweighting compared to steel. The total energy required includes processing, chemical, thermal, and energy losses during the process [75,77,78,79]. Moreover, the extraction and processing of minerals cause adverse environmental impacts, such as high energy consumption, non-renewable-resource depletion, toxic emissions, and ecosystem degradation [80,81]. Figure 3b shows the global CO_2_ emission during materials’ production. The overall emissions generated by bio-based raw materials are generally considered low, and the carbon footprints of many bio-based plastics indicate an effective strategy to get on track with the Sustainable Development Scenario (SDS).

**Figure 4:**
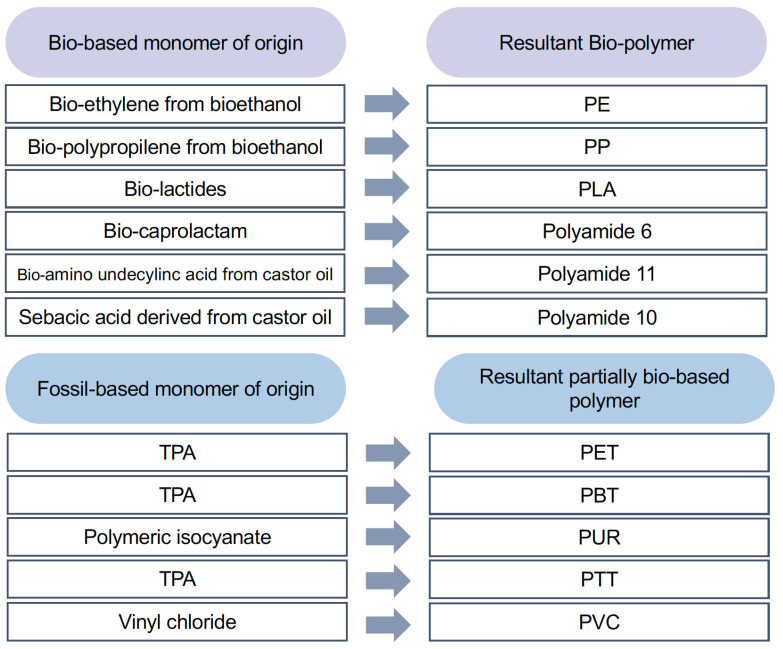
Bio-based and partially bio-based polymers suitable for automotive applications. PBS: poly(butylene succinate), PBT: polybutylene terephthalate, PLA: poly(lactic acid), PE: polyethylene, PET: poly(ethylene terephthalic acid), PP: polypropylene, PTT: polytrimethylene terephthalate, PUR: polyurethanes, PVC: polyvinyl chloride, TPA: thermoplastic polyamide.

Consequently, bio-based and partially bio-based polymers, produced from monomers such as bio-ethylene glycol, bio-ethylene, sebacic acid, and lactic acid, are good candidates for automotive applications. Bio-based and partially bio-based polymers presented in Figure 4 are some of the most suitable alternatives to traditional petroleum-based plastics for automotive applications [82,83,84,85]. Even though fossil-based monomers are coupled with bio-based monomers to make partially bio-based polymers, the carbon emission savings for bio-based polymers over fossil-derived versions are cost-effective [82]. Their strength, stiffness, and toughness are comparable to those of the petrochemical-derived ones, but some of them can be compostable. The carbon footprints of the partially bio-based polymers listed in Figure 4 are affected by their degree of bio-content. Moreover, the trend is moving from partially bio-based to fully bio-based content to minimize contamination.

### 3.2. Challenges for the Reuse of Automotive Plastic Waste

Several challenges remain that prevent the complete success of automotive plastic waste recycling for reusing. During the mechanical-recycling process, plastics endure high temperature and shear forces that could lead to thermal, thermo-mechanical, and thermo-oxidative degradation of the polymer itself and the additives present in its formulation. As a result, compounds with lower molecular weight and boiling points, capable of volatilizing and increasing contamination, accumulate and may result in reduced-quality recycled plastics [86]. For instance, recycled automotive polypropylene produces an unpleasant odor due to the oxidation of the saturated polyolefins; the autoxidation of their additives generates volatile compounds such as benzene and phenolic derivatives. Consequently, indoor automotive applications may not use post-consumer PP recycled pellets [87]. Nevertheless, catalytic pyrolysis of tires and other automotive plastics may help produce aromatic hydrocarbons valuable to produce liquid fuels [88].

Additional challenges include the lack of government recycling policies, competitively priced virgin materials, inadequate labeling, and sorting technologies, concerns with recycled plastic efficacy and appearance, limited market applications for recycled plastics, low value of recycled plastics, efficacy concerns, and even the requirement of high-volume recycling facilities. For example, using waste automobile bumpers (WAB) as a matrix and sugarcane skin flour as a filler makes a wood plastic composite with good mechanical properties but inferior to the original materials, which makes it necessary to determine their potential usages other than automotive applications [89]. Additionally, it would be necessary to have specific methods for separating and recycling automotive hybrid metal–carbon fiber structures. The process should require a step to separate metal and carbon fibers into two pure individual materials [90]. Additionally, specific processes would be needed whenever different fibers and adhesives are used to manufacture composites.

Car manufacturers are actively engaged in the recycling process because they would potentially benefit from the recycled products. Once the car’s useful life is over, the process for the car waste produced includes dismantling, depollution, shredding, and post-shredding treatment. Sometimes, conventional methods cannot achieve recycling due to additives, flame retardants, plasticizers, stabilizers, glass fibers, and contaminants. When there is no recycling option and the value of the polymer cannot be maintained, thermal treatments such as pyrolysis and landfilling are the destination for car plastics [91,92].

Finally, despite the environmental benefits, the magnitude of the added costs of the post-consumer automotive-plastics-recycling network would not be cost-effective, mainly because a plastics-labeling format that facilitates the laborious and costly task of identifying and sorting plastic sub-components for recycling would be necessary, and regulation on this matter is lacking [93].

### 3.3. Polymers in Electric Vehicles

Electric vehicles (EVs) have become an essential alternative to sustainable development in the transport sector, as reducing CO_2_ emissions is critical to mitigating climate change. The number of electric cars in circulation in the USA and Europe has increased by 344% and 742%, respectively [94]. Electric cars can mitigate CO_2_ emissions from car travel, responsible for 24% of global CO_2_ emissions, primarily when renewable energies empower production and usage [95]. Sustainability in electric cars relies partly upon the reduction in energy consumption due to the lightning of the vehicle, which makes plastics central to the process and is something to consider when considering the disposal of all materials at the end of the life cycle [96].

Battery-powered EVs’ increased production is propelling the advancement of high-performance polymers with enhanced properties to satisfy the electric-propulsion requirements. Despite the COVID-19 crisis, the global market for EV polymers, estimated at USD 3.9 billion in 2020, is projected to reach USD 27.3 billion by 2026, growing at a compound annual growth rate (CAGR) of 38.7% [97]. This growth results from the increasing regulations over the production and use of internal combustion engine (ICE) cars and the customers’ rising demand for electric cars. Nonetheless, the high cost of electric-vehicle polymers will act as a restraint on the market. Thus, a need for technologically advanced polymers mandated by electric car manufacturers is a challenge for the market players [98].

Every year in Europe, about 6 million vehicles are decommissioned due to their end-of-life through official schemes. This waste treatment establishes a minimum reuse and recycling rate of 85% of the vehicle’s total weight, but 95% of that focuses mainly on the recovery of metals. The plastics in the automotive sector are always more complex for recovering, sorting, shredding, recycling, and reusing [91]. In addition, batteries also incorporate plastics in their construction; they have separators made of microporous material of polyethylene (PE) or polypropylene (PP), which allows the maximum passage of Li-ions and acts as safety devices when they come to overheating. There are also casing plastics in packs. Fortunately, crushing, sieving, and granulometric separation can recycle plastics from batteries [94].

Although plastics have gained attention in EV manufacturing, the weight saving of high-performance polymers and polymer composites is not the sole dependable indicator of environmental achievement [99]. Efficient energy and materials are crucial for vehicle manufacturing; the lighter the vehicle, the lower the energy consumption. Thus, high-quality plastics, bio-based plastics, and biodegradable plastics could be exploited from design, making sustainability an integral concept in EV and mobility development [100], [101]. Biopolymers raise an opportunity for environmental advancement due to their biodegradable characteristics beyond lightweighting. Diminishing oil-based plastics by switching to bio-based polymers and making plastics fully recyclable are essential areas of circular economy research in the cleantech sector, like electric vehicles [102]. Additionally, end-of-life plastics can be converted to electricity in an energy recovery process. One kilogram of plastic waste has the same heating value as 1 L of fuel oil [103]. Understanding the full scope of the use of plastics in electric cars will allow the generation of industrial ecology tools for policymaking and evaluation [104].

### 3.4. Perspectives on Circular Economy

When addressing plastic-waste management, governmental institutions should aim to establish regulations to prevent plastic disposal in landfills, and scientists are encouraged to design technologies in favor of a circular economy. The circular economy redesigns materials to align economic and environmental well-being, especially by recycling. To maintain the value of polymer materials in the value chain, allowing the reuse in the original or similar applications requires calculating the maximal environmental benefits of reducing global warming impacts and fossil resource depletion while generating monomers for upcycling value-added products [105]. Recycling allows the recovery of secondary raw materials in chemical raw materials, which can be reincluded in a closed material cycle. In automotive shredder residue, including cables, electrical harnesses, and plugs, the problem is even more remarkable because there is a wide variety of plastics, from classic pure thermoplastic polymers to crosslinked plastics, numerous mixtures, and composites with fillers. It is necessary to eliminate expensive separation processes to reduce the costs related to segregation in favor of processing mixed wastes into full-value products using extrusion, rotational molding, or hot-pressing technology [106]. Moreover, significant innovations in the plastics sector derived from biotechnological methods allow producing biofuels, biochemicals, and biopolymers [107].

Circular-economy approaches are needed for every type of plastic, especially automotive plastics. Scientists have designed valuable proposals. For example, tire rubber waste could be valorized as a cost reduction additive into different materials, such as concrete, asphalt, cement, and new polymers for 3D-printing technology [108]. However, there are still several challenges for the automotive industry, such as economic and technical barriers between the reuse of post-industrial process waste and end-of-life vehicle waste. Particularly, vehicles are not designed for dismantling, which is an expensive step. Additionally, appropriate techniques must be developed and refined to identify the most cost-efficient methods to recover sufficient volumes of plastic, and a cleaning process may be needed to remove contaminants such as additives.

## 4. Conclusions

Currently, the automotive industry is meeting specific environmental goals, from using biodegradable plastics to recycling plastic parts into new ones. The substitution of metals with plastics has contributed significantly to lightweighting and energy and emission saving, but, paradoxically, also to plastic-waste accumulation. The requirements for materials that cover the modern automotive industry’s needs are increasingly demanding. Undoubtedly, lightweighting will become an even more critical strategy as more electric and autonomous vehicles are produced because battery performance is directly related to the vehicle’s mass, hence the importance of plastics. Changing the source of plastics to raw materials from renewable resources is the logical approach to sustainability. In this context, biopolymers meet this need because they have similar structural characteristics and comparable properties as their petroleum-derived counterparts. However, several challenges remain, from the selection of plastics to the recycling of end-of-life cars. One of the biggest challenges of these biodegradable polymers will be to prevent their degradation during the car’s lifetime.

## Figures and Tables

**Table 1:** Common plastics used in a typical car.

Component	Types of Polymers
Bumpers and fascia systems	PS, ABS, PC/PBT, PP, PA, PU, TPO
Seating	ABS, PA, PP
Instrument panels	ABS, PC, ABS/PC, PP
Fuel systems	POM, PA, PBT
Under hood components	PA, PBT
Interior trim	ABS, PET, POM
Electrical components	PBT, PA
Exterior trim	PS, PVC, ABS, PA, PBT, POM, ASA
Lighting systems	PC, PBT, ABS, PMMA
Upholstery	ABS, PU
Liquid reservoirs, cooling, battery carriers	PA
Wheel covers	ABS
Body parts	ABS
Tires	PA
Parts of engine	PA, phenolic resins

ABS (acryl butadiene styrene), ASA (acrylonitrile styrene acrylate), PA (polyamide), PBT (polybutylene terephthalate), PC (polycarbonate), PET (polyethylene terephthalate), PMMA (polymethyl methacrylate), POM (polyoxymethylene), PP (polypropylene), PS (polystyrene), PU (polyurethane), TPO (thermoplastic polyolefins).

**Table 2:** Automotive use of natural-fiber-reinforced polymer composites.

Manufacturer	Parts
Audi	Seat back, side, and back door panel, boot lining, hat rack, spare-tire lining
Citroen	Interior door paneling
BMW	Door panels, headliner panel, boot lining, seat back, noise insulation panels, molded foot well lining
Lotus	Body panels, spoiler, seats, interior carpets
Fiat	Door panel
Opel	Instrumental panel, headliner panel, door panels, pillar cover panel
Peugeot	Front and rear door panels
Rover	Insulation, rear storage shelf/panel
Toyota	Door panels, seat backs, floor mats, spare tire cover
Volkswagen	Door panel, seat back, boot-lid finish panel, boot-liner
Mitsubishi	Cargo area floor, door panels, instrumental panels
Daimler-Benz	Door panels, windshield/dashboard, business table, pillar cover panel, glove box, instrumental panel support, insultation, molding rod/apertures, seat backrest panel, trunk panel, seat surface/backrest, internal engine cover, engine insulation, sun visor, bumper, wheel box, roof cover
Honda	Cargo area
Volvo	Seat padding, natural foams, cargo floor tray
General Motors	Seat backs, cargo area floor
Saturn	Package trays and door panel
Ford	Floor trays, door panels, B-piller, boot liner

**Table 3:** Impact of recycling polymers.

Recycled Polymer	Estimated Cost ^a^	Impact on the Environment ^b^	Efficiency of the Recycling Process ^c^
ABS (general-purpose and impact-modified, injectable)	+	+	+ + +
ABS + PVC, ABS + PC (flame-retardant)	+	+ +	+
PA66 (flame-retardant)	+ +	+ + +	+
PA410 (impact-modified)	+ + +	+ +	+
PA + ABS, PA + PPE (injectable)	+ +	+ + +	+
PA66–40 mineral-filled	+	+ +	+
PBT (general-purpose, injectable)	+	+ +	+
PBT + PC (flame-retardant)	+	+ + +	+
PC + PMMA (flame-retardant)	+ +	+ +	+
PP20Talc	+	+	+
PP (impact-modified, UV-stabilized, flame-retardant)	+	+	+ +

+, low. + +, medium. + + +, high. a, recycling cost in USD/Kg directly attributed to the embodied energy required for recycling. b, CO_2_-equivalent mass of greenhouse gases (kg CO_2_) produced and released into the atmosphere by recycling one kg of the material. c, estimation of the real recycling fraction in the current supply.

## Data Availability

Data presented in this study is available upon request from the corresponding author.

## Abbreviation

ABS	Acrylonitrile butadiene styrene
ASA	Acrylonitrile styrene acrylate
Bio-PA	Bio-polyamides
CAGR	Compound annual growth rate
EV	Electric vehicle
HDPE	High-density polyethylene
ICE	Internal combustion engine
IEA	International Energy Agency
LDPE	Low-density polyethylene
ABS-g-MA	Maleic anhydride-grafted acrylonitrile butadiene styrene
PL	Photoluminescence
PBS	Poly butylene succinate
PA	Polyamide
PBT	Polybutylene terephthalate
PC	Polycarbonate
PE	Polyethylene
PET	Polyethylene terephthalate
PHA	Polyhydroxyalkanoates
PHB	Polyhydroxybutyrate
PLA	Polylactide acid
PMMA	Polymethyl methacrylate
POM	Polyoxymethylene
PPS	Polyphenylene sulfide
PP	Polypropylene
PS	Polystyrene
PTT	Polytrimethylene terephthalate
PU	Polyurethane
PUR	Polyurethanes
PVC	Polyvinylchloride
SMA	Styrene maleic anhydride
SDS	Sustainable Development Scenario
KIT	The Karlsruhe Institute of Technology
TPA	Thermoplastic polyamide
TPO	Thermoplastic polyolefins
WAB	Waste automobile bumper
